# A Neurosyphilis Case Mimicking Herpes Simplex Virus Encephalitis in an African American Male

**DOI:** 10.7759/cureus.59093

**Published:** 2024-04-26

**Authors:** Osama Elkhider, Monzer Abdalla, Omer A Ibrahim, Abdurrahman Mustafa, Shamsa Abdelatif

**Affiliations:** 1 Internal Medicine, Medical College of Wisconsin, Milwaukee, USA; 2 Internal Medicine, Ascension Saint Francis Hospital, Evanston, USA; 3 Internal Medicine, Omdurman Military Hospital, Khartoum, SDN; 4 Internal Medicine, University of Medical Sciences and Technology, Khartoum, SDN

**Keywords:** sexually transmitted infection (sti), altered mental state, encephalitis, neurosyphilis, herpes simplex

## Abstract

Neurosyphilis is one form of a multisystemic sexually transmitted disease caused by *Treponema pallidum*. Although typical presentations of neurosyphilis have become less common in the post-antibiotic era, a rising trend of atypical presentations can mimic other diagnoses like herpes simplex and autoimmune encephalitis. In this case, we diagnosed neurosyphilis in a patient with clinical and radiological features similar to herpes simplex encephalitis. We emphasize the need for a diagnostic approach combining imaging namely MRI, lumbar puncture, and use of treponemal and non-treponemal tests so that neurosyphilis cases with atypical neuroimaging findings are not overlooked.

## Introduction

Syphilis is a bacterial multisystemic sexually transmitted disease caused by *Treponema pallidum* [1,2]. It is classified into congenital syphilis or acquired [3]. Based on the duration from the time of infection to the appearance of symptoms and signs, syphilis can be divided into early and late, sometimes overlapping stages. Early syphilis (within one year), which includes primary syphilis heralded by painless syphilitic ulcer known as chancre after an incubation of 10-90 days, secondary which includes multisystemic involvement caused by bacteremia and early latent (non-primary non-secondary early syphilis) in which patient serological tests are positive with no clinically evident infection [3]. Late syphilis includes late latent (within more than one year from infection) and tertiary syphilis. Early and secondary syphilis are the most infectious stages, usually through sexual contact, while at late stages, transmission is usually vertically or through organ transplants [3].

Neurosyphilis is the tertiary stage of syphilis, where the central nervous system is involved. However, it can manifest at any stage of the disease. Neurosyphilis can be further subdivided into a meningovascular form, which may present as a stroke and meningitis, and a parenchymal form, which may present as paresis, neurocognitive decline, and dementia [4]. The spectrum of presentations extends beyond clinical signs and symptoms to include imaging findings, which could be difficult to distinguish from other common diseases like viral encephalitis solely on a radiological basis [5].

Significant numbers of patients with neurosyphilis are reported to have normal neuroimaging, including CT, MRI, and angiography, as well as nonspecific cerebral atrophy. Other common findings include infarctions, white matter lesions, myelitis, cerebral atrophy, cord atrophy, T-2 weighted hyperintensities of the spinal cord, leptomeningeal enhancement signifying meningitis, leptomeningeal gummata, gummatous periostitis, and meningoneuritis with cranial nerve neuropathies [4].

## Case presentation

A 61-year-old African American male with no significant known past medical history was brought to the hospital after being found unresponsive on the floor of his apartment. The patient previously resided independently and was last seen in a normal state the day before the presentation. On presentation, he had altered consciousness and was unresponsive, grimacing only to pain. His vital signs are within normal limits. His Glasgow Coma Scale (GCS) was 8. The neurological exam was limited because of his altered mental status. However, there was no neck stiffness or facial asymmetry, and pupils were equal in size and reactive to light. The tone and reflexes were normal, and no focal weakness was noted. His initial laboratory results are shown in Table 1.

**Table 1:** Hematology and chemical laboratory results WBCs: white blood cells; CRP: C reactive protein; CK: creatine kinase; K: potassium; Na: sodium; Ca: calcium; BUN: blood urea nitrogen; AST: aspartate transaminase; ALT: alanine transaminase; ALP: alkaline phosphatase; TSH: thyroid-stimulating hormone

Lab test	Patient result	Reference range
WBCs	17x10^9^/L	3.9- 11.2x10^9^/L
Procalcitonin	2.6 ng/ml	0.2-0.49 ng/ml
CRP	14 mg/dL	<1.0 mg/dL
CK	2300 IU/L	30.0 – 223.0 IU/L
K^+^	3.2 mmol/L	3.5 – 5.2 mmol/L
Na^+^	138 mmol/L	133-144 mmol/L
Ca^+2^	8.9 mg/dL	8.6-10.3 mg/dL
Serum glucose	209 mg/dL	70-99 mg/dL
BUN	12 mg/dL	7-25 mg/dL
Creatinine	1.09 mg/dL	0.6-1.3 mg/dL
AST	66 IU/L	13-39 IU/L
ALT	28 IU/L	7-52 IU/L
ALP	63 IU/L	40-129 IU/L
Total Bilirubin	1.8 mg/dL	0-1 mg/dL
Ammonia	31 µmol/L	16-52 µmol/L
TSH	3 mIU/L	0.5-5 mIU/L

CT of the head and CT angiogram of the head and neck showed no hemorrhage, midline shift, or mass effect. A normal ventricular system and gray-white matter junction were identified. There were periventricular microangiopathic chronic white matter ischemic changes, as well as atherosclerotic calcifications in the vertebrobasilar system and the intracranial portions of the internal carotid arteries. With the presence of leukocytosis and in the absence of an obvious source of infection, a lumbar puncture was done to obtain cerebrospinal fluid (CSF). The patient was then treated empirically for possible meningoencephalitis with an antibiotic and an antiviral agent. Unfortunately, no remarkable improvement was noted while on this regimen. The CSF analysis results are shown in Table 2.

**Table 2:** Cerebrospinal fluid chemical and hematological results WBCs: white blood cells, RBCs: red blood cells

Lab test	Patient’s result	Reference range
Glucose	47 mg/dL	40-70 mg/dL
Protein	103 mg/dL	15-45 mg/dL
RBCs	1/mm^3^	0-10/mm^3^
WBCs	35/mm^3^	0-5/mm^3^
Lymphocytes	94%	
Neutrophils	2%	
Monocytes	4%	

**Figure 1:**
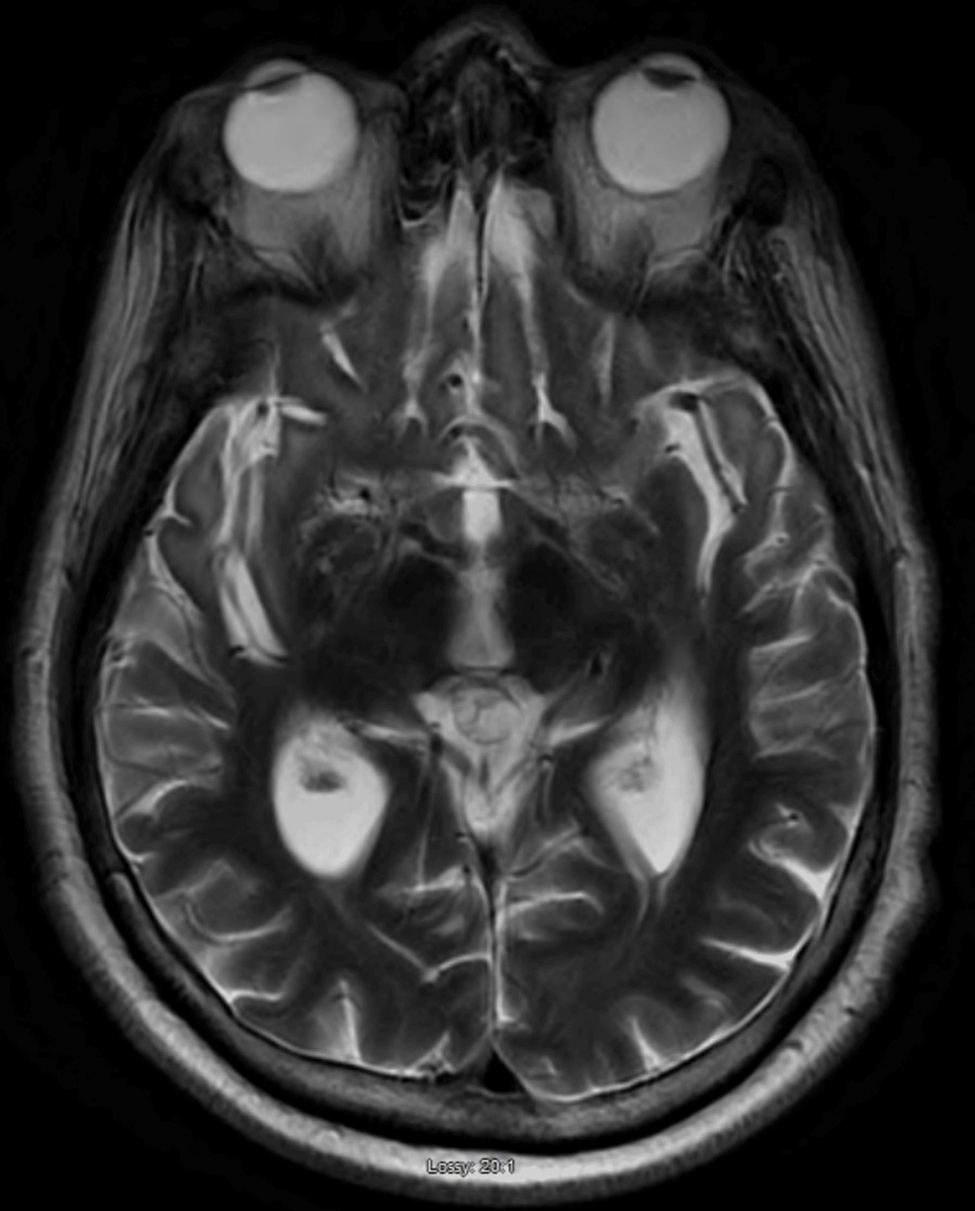
MRI with diffuse cerebral volume loss

**Figure 2:**
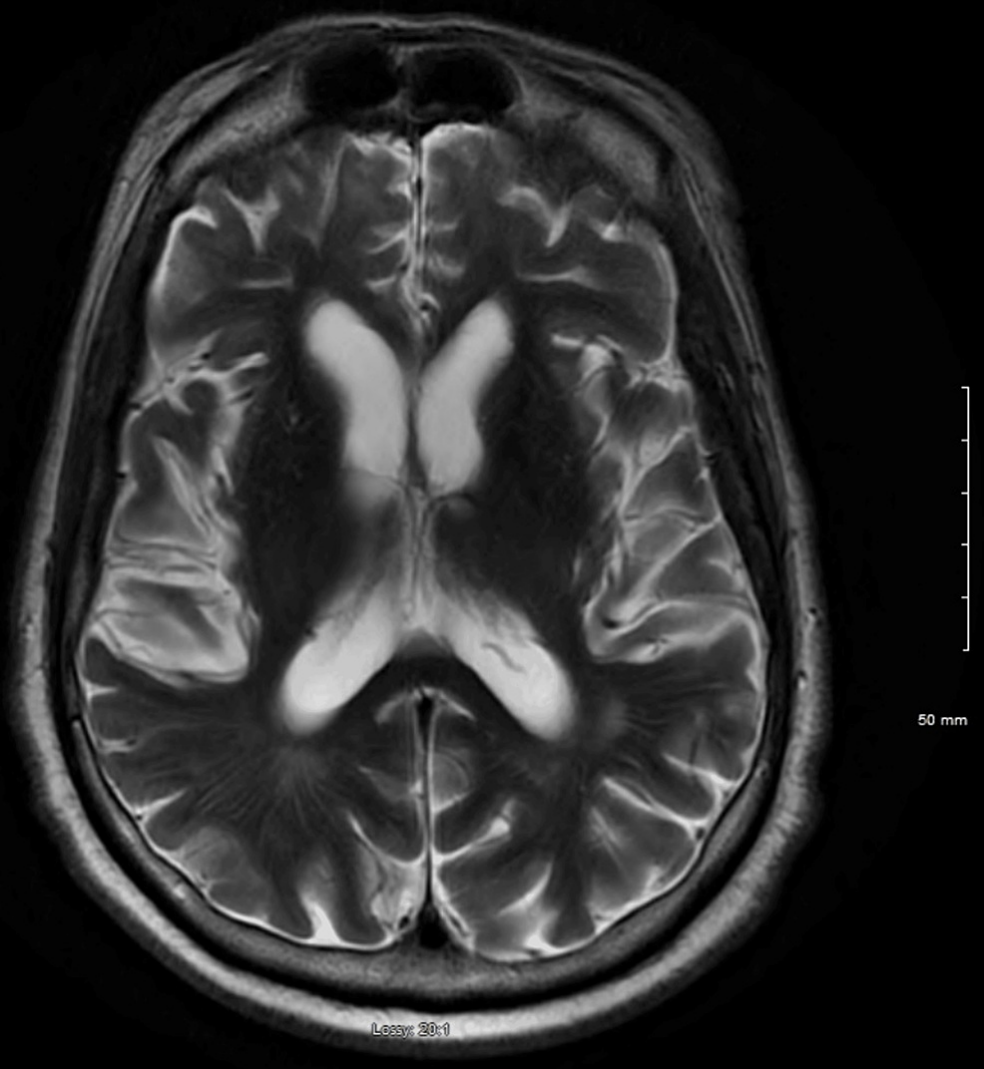
T2 weighted MRI brain with hyperintensity in temporal lobes

**Figure 3:**
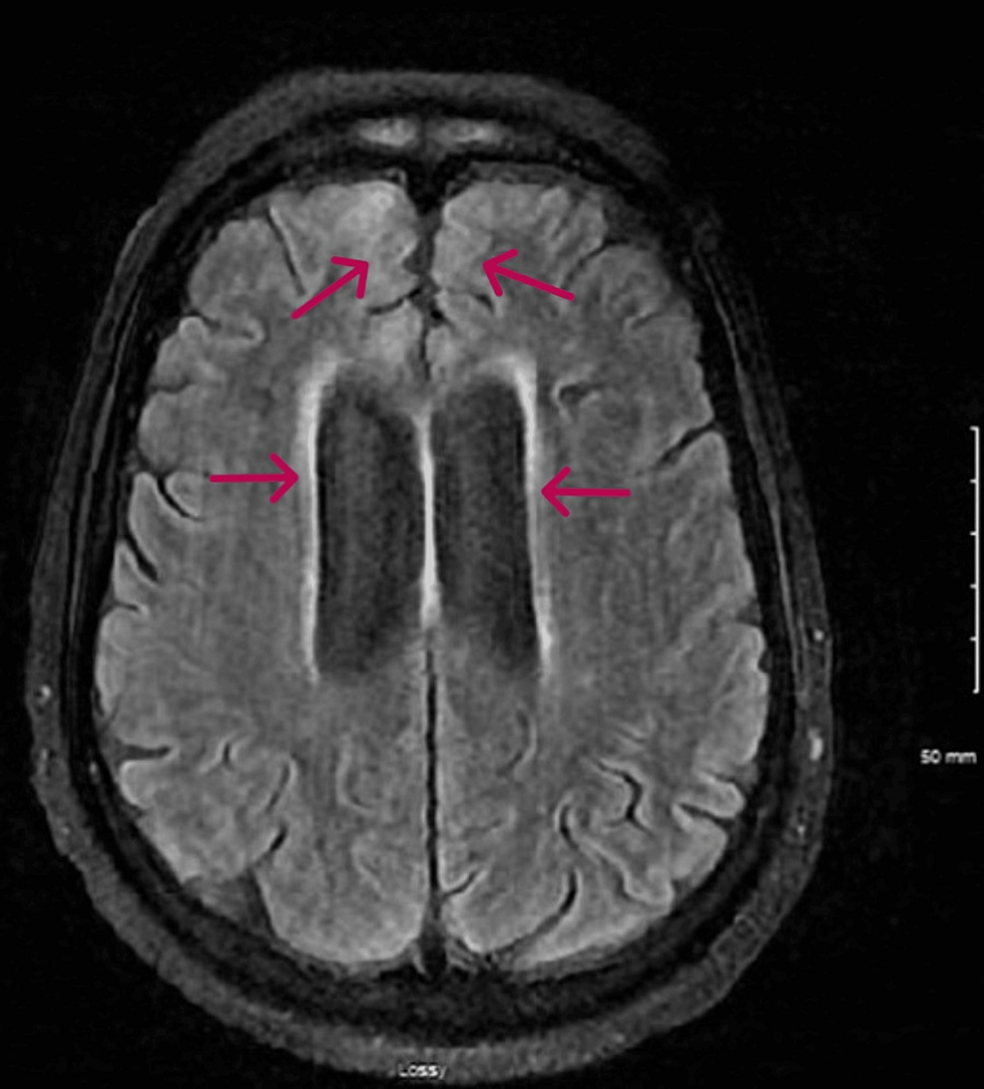
MRI Brain with persistent FLAIR signal hyperintensity in temporal lobes, particularly in the anterior horns, in the insular cortices, and the anterior inferior frontal lobes FLAIR: fluid-attenuated inversion recovery

After the initial CSF results, a brain MRI scan was obtained which showed mild diffuse cerebral atrophy (Figure 1), persistent T2 hyperintensity in temporal lobes (Figure 2), and persistent fluid-attenuated inversion recovery (FLAIR) signal hyperintensity in temporal lobes, particularly in the anterior horns, the insular cortices, and the anterior inferior frontal lobes (Figure 3).

Two days after the MRI results, the Venereal Disease Research Laboratory (VDRL) test in the CSF came reactive, while the varicella-zoster virus and herpes simplex virus types 1 and 2 by polymerase chain reaction (PCR) from the CSF were not detected.

Given the above results, a rapid plasma reagin (RPR) was obtained and was reactive in his serum. The HIV test came back negative. Electroencephalogram (EEG) showed diffuse slowing of background activity, and no focal or generalized epileptiform discharges were noted.

The patient was started on penicillin G for syphilis for 14 days. The patient's leukocytosis normalized in the blood. Repeat CSF analysis showed normalization of white cell counts and reduction of CSF proteins to 90 mg/dL. His mental status showed some improvement with a GCS of 12. Still, he continued to be disoriented with the expression of nonsensical speech. He remained functionally dependent for over six weeks with no improvement in his cognition till his discharge to a skilled nursing facility. MRI was not repeated after the completion of therapy but CSF analysis was repeated and showed improvement in lower proteins and WBCs.

## Discussion

Even though the post-penicillin era showed a significant decline in syphilis cases, growing numbers of cases are being diagnosed, including neurosyphilis, especially among people living with HIV and other immunocompromised individuals [6]. Our patient’s clinical presentation was quite interesting as his mental status deterioration had an acute course rather than the known prolonged course of cognitive changes seen in neurosyphilis. Whether he did not have cognitive/memory issues or minimal cognitive changes that the people around him could not recognize is unclear.

Given his acute course, our thoughts were toward the acute conditions that could cause acute encephalitis, including strokes, viral, bacterial meningoencephalitis, and toxic and metabolic causes of acute encephalopathy. His CT scan of the head and the CT angiogram were unremarkable, which is not uncommon for patients with neurosyphilis [4]. However, his MRI findings showed persistent FLAIR signal hyperintensity in temporal lobes, particularly in the anterior horns, which are atypical for neurosyphilis [4] but rather suggestive of herpes simplex encephalitis. The results of the positive VDRL test in the CSF and his reactive RPR were surprising, given the clinical and radiological presentation of the patient.

The patient was initially treated empirically with acyclovir and ceftriaxone, which, unfortunately, were not the drugs of choice for syphilis. The patient received the appropriate treatment after the results of the VDRL in the CSF and RPR became available, which resulted in a two-day delay in treatment. Unfortunately, despite treating the patient with a 14-day course of intravenous penicillin G, his mental status did not improve optimally, and he remained with a significant residual neurocognitive deficit.

Our case supports the few case reports in the literature that describe similar clinical and radiological presentations of neurosyphilis [7]. We believe that it is important to recognize this pattern of presentation to avoid misdiagnosing and/or treatment delays. We suggest including a screening RPR for all patients with acute encephalopathy and testing for VDRL in the CSF, even if the MRI picture is consistent with herpes infection [3].

## Conclusions

Neuroimaging findings in neurosyphilis are not limited to the typical findings and can extend to findings that mimic those seen in a patient with herpes simplex encephalitis. Neurosyphilis should be considered in the differential diagnosis of the patient with acute encephalopathy, and physicians should have a low threshold for testing for syphilis to avoid misdiagnosing such atypical presentations of neurosyphilis.
